# Nasal Angiomyolipoma (AML) Mimicking Juvenile Nasopharyngeal Angiofibroma 

**Published:** 2019-05

**Authors:** Saiful-Azhar Ameen, Husain Salina, Farah-Dayana Zahedi, Sabir-Husin-Athar Primuharsa-Putra, Noraidah Masir

**Affiliations:** 1 *Department of Otorhinolaryngology-Head and Neck Surgery, Universiti Kebangsaan Malaysia Medical Centre, Kuala Lumpur, Malaysia.*; 2 *Ear, Nose and Throat - Head & Neck Consultant Clinic, KPJ Seremban Specialist Hospital/ KPJ Healthcare University College, Negeri Sembilan, Malaysia.*; 3 *Department of Pathology, * *Universiti * *Kebangsaan Malaysia Medical Centre, Kuala Lumpur, Malaysia.*

**Keywords:** Epistaxis, Endoscopic sinus surgery, Nasal angiomyolipoma, Perivascular epithelioid cell tumor (PEComa), Juvenile nasopharyngeal angiofibroma

## Abstract

**Introduction:**

Angiomyolipoma (AML), a benign mesenchymal tumor that commonly arises from the kidney, may be associated with tuberous sclerosis complex and perivascular epithelioid cell tumors (PEComas). Nasal angiomyolipoma is very rare and usually occurs in elderly individuals with epistaxis and nasal obstruction.

**Case Report:**

We report a rare case of nasal angiomyolipoma in a young male. To the best of our knowledge, this is the first documented case of angiomyolipoma originating from the posterior end of the inferior turbinate, clinically mimicking juvenile nasopharyngeal angiofibroma (JNA). The tumor was removed completely via coblator-assisted endoscopic sinus surgery. The patient was asymptomatic at a 2-year follow-up.

**Conclusion:**

Nasal AML located in the posterior nasal cavity in a male patient can mimic the presentation of JNA. A computed tomography scan of the paranasal sinuses played an important role in differentiating nasal AML from JNA. The coblator-assisted endoscopic technique is useful in controlling intraoperative hemostasis in the removal of a suspicious vascular tumor.

## Introduction

Angiomyolipoma (AML) is a benign mesenchymal tumor with the characteristic composition of mature adipose tissue, smooth muscle cells, and abnormally thick-walled blood vessels that are present in varying proportions (1). Most AMLs arise from the kidney, less often in the liver, and rarely in other anatomic sites. Only a few cases in the mediastinum, heart, spermatic cord, vaginal wall, fallopian tube, oral cavity, pharynx, and skin have been reported (2). Intranasal localization of AML is exceedingly rare. AML occurs in two distinct clinical settings: sporadic, or in association with the tuberous sclerosis complex (TSC). About 80% of the cases of AML are classified as isolated or sporadic; however, among those who inherit TSC, 80% will be affected by renal AMLs (3). Most cases of angiomyolipoma are benign, although a malignant form of angiomyolipoma has also been documented (4). Nasal AML tends to occur in older men, and is not associated with TSC (5). Nasal AMLs usually present with nasal obstruction and epistaxis (6,7–10).

Juvenile nasopharyngeal angiofibroma (JNA) is a highly vascular tumor, which primarily affects adolescent males (11). JNA patients present with unilateral nasal obstruction, recurrent epistaxis, and nasopharyngeal mass (12).We report an extremely rare case of intranasal angiomyolipoma with a clinical presentation mimicking JNA in a 28-year-old male. To the best of our knowledge, this is the first case reported on an angiomyolipoma arising from the posterior end of the inferior turbinate, and removal of the tumor using a coblator-assisted endoscopic technique.

## Case Report

**Fig 1:**
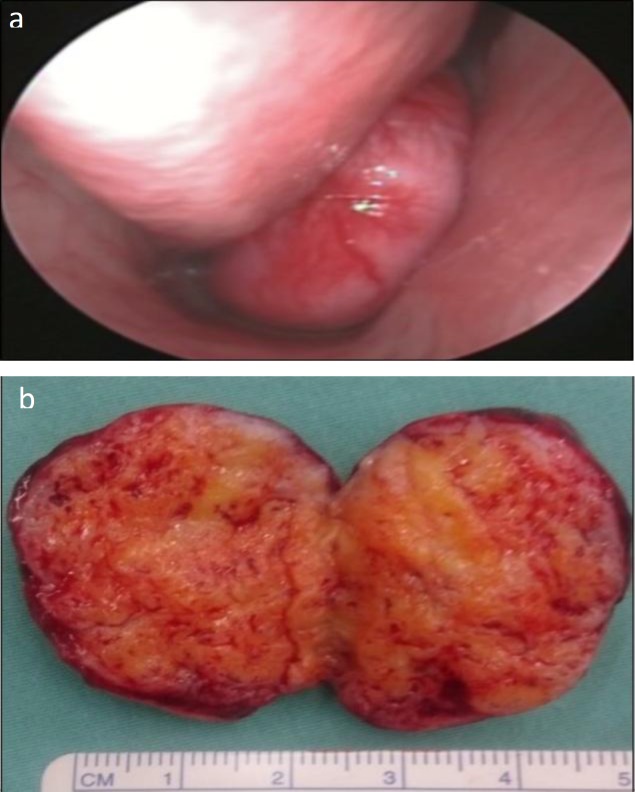
a) Reddish mass with prominent blood vessels arising from posterior end of right inferior turbinate; b) Macroscopic appearance of the tumor

A 28-year-old male presented with a history of intermittent mild-to-moderate epistaxis for 14 years. He experienced 3–4 episodes of epistaxis per year, which stopped spontaneously. The symptom was associated with progressively worsening right nasal obstruction, hyposmia, and headache. He had no neck swelling or stigmata of tuberous sclerosis.Nasal endoscopy showed a reddish mass, with prominent blood vessels arising from the right lateral wall of the post-nasal space extending to the nasopharynx (Fig1a). 

**Fig 2:**
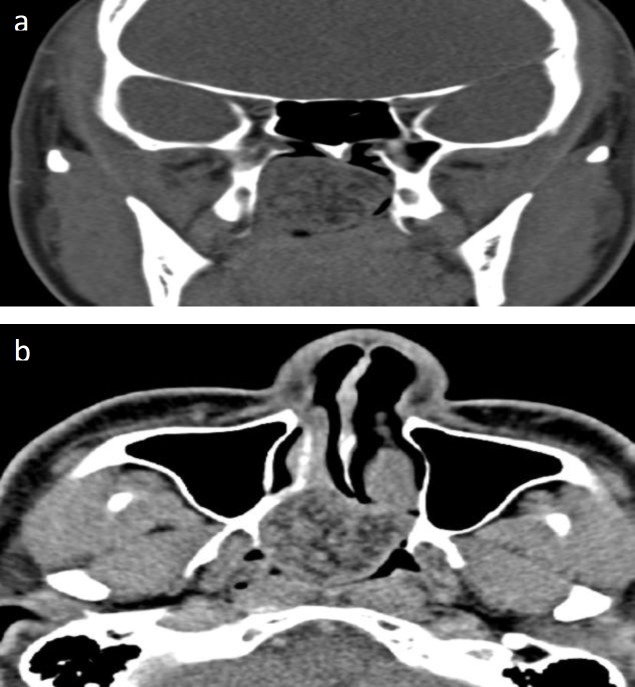
a) Axial and b) coronal view a CT scan of the paranasal sinuses showing a lobulated mass arising from the posterior end of the right inferior turbinate extending to the nasopharynx

A computed tomography (CT) scan of the paranasal sinuses showed a lobulated non-enhancing mass at the right posterior nasal space arising from right posterior end of the inferior turbinate (Fig.2).

Coblator-assisted endoscopic removal of the tumor was performed under general anesthesia. The lesion was vascular, and copious bleeding was encountered during surgery. Intraoperative blood loss was 200 ml. The patient was discharged 2 days post surgery.

**Fig 3:**
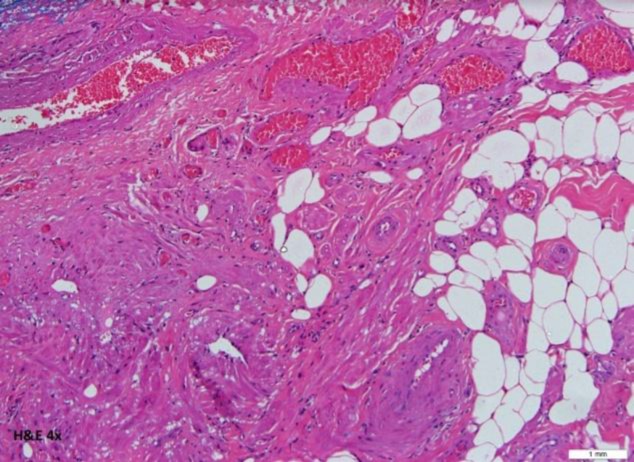
Angiomyolipoma. Lesion shows an admixture of haphazardly arranged mature adipose tissue, smooth muscle fibers and thick-walled blood vessels. The lesion is partly lined by the respiratory epithelium

Grossly, the tumor was a well-circumscribed homogenous whitish tissue measuring 4.0×3.0×2.5 cm, with numerous intervening small blood vessels (Fig.1b). Histologically, the section of the lesion shows an admixture of haphazardly arranged mature adipose tissue, smooth muscle fibers and thick-walled blood vessels. The lesion is partly lined by the respiratory epithelium (Fig.3).

 The intervening blood vessels are composed of arteries, arterioles, capillaries, venules, and veins. No atypical cell or evidence of malignancy was seen. Immunohistochemically, the endothelium of the vessel was positive for CD31, the elastic tissue of the vessel wall was positive for elastic Van Gieson (EVG), and the smooth muscle bundle fibers were positive for smooth muscle actin (SMA) and desmin. The melanocyte marker HMB-45 was negative. The histopathology examination was consistent with nasal angiomyolipoma.

At a 2-year follow-up, the patient was asymptomatic, and endoscopic examination showed no recurrence of the tumor.

## Discussion

Watanabe suggested that nasal AML is termed as ‘mucocutaneous angiomyolipoma’ because the clinicopathologic features of AML of the nasal cavity are common to those of AML arising in the skin and oral and pharyngeal mucosa and are distinct from renal and hepatic AML (13). AML in the nasal cavity includes only mature smooth muscle cells and is negative to HMB-45 melanoma specific antigens on immunohistochemical staining, unlike renal AML (6).

Although AML has been known for quite some time, it has only recently become clear that it is part of a family of tumors that may arise in essentially any location in the body. The members of this remarkable family of tumors, now known as perivascular epithelioid cell tumors (PEComas), are characterized by the distinctive morphology of their constituent PEC and by their unusual “myomelanocytic” immunophenotype (14). The term PEComa, first coined by Bonetti et al. (15), is currently defined by WHO as a mesenchymal tumor composed of distinctive perivascular epithelioid cells (16). PEComas are a family of neoplastic lesions that share overlapping morphology, immunohistochemistry, and ultrastructure and includes angiomyolipoma (AML), lymphangioleiomyomatosis (LAM), clear cell ‘sugar’ tumor (CCST) of the lung, as well as similar tumors occurring in a variety of visceral, cutaneous, and soft tissue sites throughout the body. PEComas are rare in the head and neck, and those arising in the nasal cavity and larynx are even more unusual (7). However, Tosios discourages the use of the term AML for angiomyomatous lesions with adipocytes, as there is no evidence of any relationship of such lesions with renal or retroperitoneal AML, or with tuberous sclerosis (17).To date, fewer than 20 cases of nasal AML/PEComa have been reported worldwide. Nasal cavity AML tends to occur in older men (6), while JNA affects primarily adolescent males (11). In our case, the patient had episodes of epistaxis since his adolescent years. A case of nasal AML in adolescents has not been reported before, but two cases of nasal PEComas in female adolescents have been reported (7,8). Our patient had episodes of mild-to-moderate epistaxis that mimics the presentation of JNA. However, the prolonged clinical course from presentation to diagnosis makes the diagnosis of JNA improbable, as JNA usually presents with severe epistaxis that necessitates early intervention. Histopathologically, JNAs are non-encapsulated tumors composed of a mixture of blood vessels and fibrous stroma containing blood vessels of different sizes and shapes lined by endothelial cells, but with little or no smooth muscle or elastic fibers. This structure, lacking muscles fibers, unlike the AML, contributes to the capacity of JNAs to bleed excessively after minimal manipulation, thus making their surgical management a challenge (12).

Imaging plays an important role in diagnosing a nasal mass. A CT scan is better in evaluating bony details compared with plain films and magnetic resonance imaging (MRI). In JNA, features such as anterior bowing of the posterior maxillary sinus wall (Holman-Miller sign), widening of the sphenopalatine foramen, and bony erosion are common (18). In our case, the CT scan did not show any of those findings; thus, the diagnosis of JNA is unlikely. Angioembolization prior to surgery was not performed because the preoperative CT scan showed features of non-enhancing mass and no classical features of JNA. Nasal AMLs are generally excised via endoscopic sinus surgery. In our patient, coblator-assisted endoscopic removal of the tumor was performed with moderate intraoperative bleeding. 

Gana reported a case of nasal PEComa removed en-bloc using an endoscopic diode laser with good post-operative outcome (9). Although Shang et. al. used therapeutic angioembolization in managing nasal AML, other authors reported the removal of nasal AMLs without prior angioembolization (5,6-8,10,13), and there have been no reports of excessive intraoperative bleeding in these patients. Thus, unlike JNA, angioembolization was rarely needed prior to surgical removal of nasal AML.

## Conclusion

In conclusion, nasal AML located in the posterior nasal cavity in a male patient can mimic the presentation of JNA, which is one of the most common causes of recurrent epistaxis in adolescents. It should also be considered as one of the differential diagnosis of unilateral nasal mass. A CT scan plays an important role in differentiating nasal AML from JNA. The coblator-assisted endoscopic technique is useful in controlling intraoperative hemostasis in the removal suspicious vascular tumors.
